# Mandibular Movement During Swallowing in Patients with Tinnitus: An Instrumented Case–Control Study

**DOI:** 10.3390/audiolres16020038

**Published:** 2026-03-05

**Authors:** Henri Albert Didier, Federica Di Berardino, Giorgio Lilli, Diego Zanetti, Alexander Henri Didier, Giorgio Raponi, Saverio Joshua Leone, Silvia Romano, Marco Farronato, Elisa Boccalari, Marco Serafin, Alberto Caprioglio, Dino Re, Aldo Bruno Giannì

**Affiliations:** 1 Department of Biomedical, Surgical and Dental Sciences, University of Milan, Via della Commenda 10, 20122 Milan, Italy; hdidier@email.it (H.A.D.); saverio.leone99@gmail.com (S.J.L.); silvia.romano@unimi.it (S.R.); marco.serafin@unimi.it (M.S.); alberto.caprioglio@unimi.it (A.C.); dino.re@unimi.it (D.R.); aldo.gianni@unimi.it (A.B.G.); 2 Istituto Stomatologico Italiano, 20122 Milan, Italy; 3 Department of Clinical Sciences and Community Health, University of Milan, Via della Commenda 19, 20122 Milan, Italy; federica.diberardino@unimi.it (F.D.B.); diego.zanetti.bs@gmail.com (D.Z.); 4 Audiology Unit, Department of Surgical Sciences, Fondazione IRCCS Ca’ Granda Ospedale Maggiore Policlinico, 20122 Milan, Italy; giorgio.lilli@policlinico.mi.it; 5 Department of Pharmacy, Hirslanden, 1224 Geneve, Switzerland; alexandredidi@hotmail.com; 6 Private Practice, 20124 Milan, Italy; grraponi@gmail.com; 7 Maxillo-Facial Surgery and Dental Unit, Fondazione IRCCS Ca’ Granda Ospedale Maggiore Policlinico, 20122 Milan, Italy

**Keywords:** kinesiography, swallowing, tinnitus, tongue thrust, tubal insufficiency

## Abstract

**Objectives**: This study aimed to determine whether adults with tinnitus exhibit altered phase-specific mandibular kinematics during saliva swallowing and increased prevalence of tongue thrust and Eustachian-tube insufficiency versus tinnitus-free controls. **Methods**: This was a cross-sectional case–control study including adults with tinnitus and controls. Standardized computerized kinesiography recorded three spontaneous saliva swallows per participant. Primary outcomes were opening/closing time (OCT) and post-closure stabilization time (STT); total swallowing time (SWT) was secondary. Tongue thrust (TT) and tubal insufficiency (TI) were assessed clinically. Distributional assumptions were checked with Shapiro–Wilk; between-group comparisons used two-sided Mann–Whitney U tests and Fisher’s exact tests (TT, TI). Effect sizes included rank-biserial correlation (*r*), Hodges–Lehmann median difference (Δ), and odds ratios (ORs) with 95% confidence intervals. Co-occurrence of TT and TI and their relationships with OCT, STT, and SWT were evaluated within strata (cases vs. controls) using Fisher’s exact test, *φ*, Mann–Whitney U tests, and Spearman’s *ρ*. Given the marked imbalance in age and sex between groups, unadjusted non-parametric comparisons were complemented by multivariable models with adjustment for age and sex. An omnibus non-parametric combination test summarized case–control differences across OCT, STT, and SWT. **Results**: Statistical analysis was performed on 77 cases with tinnitus and 78 controls. Tinnitus cases showed longer OCT (1.75 ± 0.92 vs. 1.12 ± 0.62 s; *p* < 0.001; *r* ≈ 0.40; Δ ≈ +0.60 s) and STT (1.44 ± 0.88 vs. 0.84 ± 0.62 s; *p* < 0.001; *r* ≈ 0.42; Δ ≈ +0.60 s), while SWT differed modestly and was not significant (2.75 ± 0.69 vs. 2.57 ± 0.65 s; *p* = 0.115; *r* ≈ 0.15; Δ ≈ +0.18 s). TT was more frequent in cases (18.2%) than controls (6.4%; OR = 3.05, 95% CI 1.08–8.61; *p* = 0.029), whereas TI occurred in 16.9% of cases and 0% of controls (corrected OR = 32.85, 95% CI 1.92–563.49; *p* < 0.001). Within tinnitus cases, TT and TI did not show meaningful co-occurrence (*φ* ≈ −0.03; *p* = 1.00). TT+ tinnitus patients exhibited markedly prolonged OCT compared with TT− (median 2.22 vs. 1.45 s; Δ ≈ +0.88 s; *r* ≈ 0.60; *p* < 0.001), whereas STT and SWT were minimally affected; TI was not materially associated with any swallowing-time parameter. Spearman analyses confirmed a moderate monotonic association between TT and OCT in tinnitus cases (*ρ* ≈ 0.40; *p* < 0.001), with all other correlations small and clinically negligible. Age- and sex-adjusted analyses confirmed longer OCT and STT in tinnitus cases, whereas SWT remained non-significant; TT and TI also remained more frequent in cases after adjustment. The omnibus test indicated a clear global separation between groups across OCT, STT, and SWT (permutation *p* < 0.001). **Conclusions**: Adults with tinnitus exhibit a distinct swallowing signature characterized by prolonged OCT and STT, together with higher prevalence of TT and TI. TT in tinnitus patients is specifically linked to a pronounced prolongation of OCT, while STT and SWT remain largely unchanged, and TI shows no relevant impact on kinematic indices.

## 1. Introduction

Tinnitus is a prevalent and often disabling percept defined by the experience of sound in the absence of an external source [1]. Its mechanisms are heterogeneous, spanning peripheral, brainstem, and cortical processes, and growing evidence indicates that non-auditory somatosensory inputs can modulate auditory activity through well-described convergence within trigeminal–cochlear and cervical–auditory pathways [2]. Among these, hearing loss is described as one of the most common contributors to tinnitus, and evidence that auditory rehabilitation can influence symptom severity supports a relevant role for peripheral mechanisms in its pathophysiology [3]. From a somatosensory perspective, orofacial and oropharyngeal signals may shape symptom expression through interactions with auditory circuits and modulation of middle-ear mechanics [4]. Assessing whether objectively measurable features of these systems differ in individuals with tinnitus may therefore help delineate pathophysiology [5].

Swallowing offers a particularly informative window into this problem. It is a high-frequency, tightly coordinated motor behavior that recruits mandibular elevators and depressors, tongue musculature, soft palate and pharyngeal constrictors, and reflexive adjustments of the Eustachian tube [6]. Subtle inefficiencies in this network may manifest as altered mandibular kinematics during the opening/closing phase of the swallow and during the subsequent stabilization period when the mandible settles into occlusal contact [7]. These timing parameters provide time-resolved indices of neuromuscular coordination and occlusal settling. In addition, two clinically relevant signs address complementary structural–functional domains with potential somatosensory links to tinnitus perception: tongue thrust and tubal insufficiency [8,9].

Despite the biological plausibility of these links, instrument-based comparisons between individuals with tinnitus and those without it remain scarce [10,11]. Much of the existing literature relies on symptom inventories, broad audiologic endpoints, or global measures of jaw function, which are essential for clinical characterization but may be insensitive to phase-specific deviations in swallowing dynamics or to discrete signs such as tongue thrust and tubal insufficiency [12,13]. As a result, it is unclear whether tinnitus is associated with quantifiable alterations in swallowing-phase timing or with an increased prevalence of these signs, and whether any single parameter is informative on its own or whether a coordinated pattern across multiple readouts better captures the phenotype [14].

The present study addresses this gap by examining whether individuals with tinnitus differ from controls in objective, kinematics-based indices of saliva swallowing and in the occurrence of tongue thrust and tubal insufficiency. If robust differences are confirmed, this would reinforce a role for somatosensory influences tied to orofacial–oropharyngeal control in tinnitus. Conversely, null findings would delimit the relevance of swallowing-phase kinematics and the assessed clinical signs, motivating future work on other mechanistic pathways. In either scenario, a systematic, instrument-anchored comparison promises to move the field beyond symptom correlation toward mechanism-informed, assessment based on objective kinematic measures.

## 2. Materials and Methods

The present study was conducted in accordance with the STROBE guidelines and the principles of the Declaration of Helsinki [15,16]. The protocol was approved by the local ethics committee of Fondazione IRCCS Ca’ Granda Ospedale Maggiore Policlinico (Protocol No. 653) and participants provided written informed consent prior to enrollment. All data were anonymized and stored under a coded identifier.

This was a cross-sectional case–control study comparing adults with tinnitus (77 cases) to controls without tinnitus (78 controls) using standardized, instrument-based assessment of spontaneous saliva swallowing. Participants were recruited consecutively from the Audiology and Odontostomatology Units of Fondazione IRCCS Ca’ Granda (Ospedale Maggiore Policlinico) and the Istituto Stomatologico Italiano, Milan, Italy. Eligibility required the ability to comply with kinesiographic recording and to perform spontaneous swallowing; exclusion criteria comprised pulsatile tinnitus, acute upper–airway infections, recent orofacial surgery or trauma, active dental pain precluding occlusal contact, and any neurological condition known to impair swallowing or jaw motor control. Tinnitus was determined at screening by clinician evaluation corroborated by the participant’s report; controls denied current or past tinnitus. All cases presented chronic tinnitus of at least 6 months’ duration, which was continuous rather than intermittent, and somatically modulable at clinical examination. Tinnitus severity was assessed using the Tinnitus Handicap Inventory (THI), and only patients with THI scores below 60 (mild-to-moderate handicap) were included to reduce heterogeneity related to severe distress or disability that could interfere with task performance. Tinnitus laterality was not systematically recorded. All cases included in the study had undergone an audiological/ENT evaluation to exclude specific otologic and neurologic causes and were identified as having idiopathic tinnitus. Moreover, participants provided written informed consent prior to any procedure.

Swallowing was acquired with computerized kinesiography (K7/CMS; Myotronics/Normed Inc., Tukwila, WA, USA), which records mandibular motion in real time with high temporal resolution [17]. Participants were seated upright with the head in natural position and were instructed to relax and allow the mandible to assume its physiological rest position. They were informed that mandibular movement would be observed for several minutes and that spontaneous saliva swallowing was permitted during the observation period. Immediately after each swallow, participants were asked to close the mouth and bring teeth into contact in centric occlusion. A calibration routine was performed according to the manufacturer’s instructions to ensure accurate registration of vertical motion relative to intercuspation. To avoid cueing effects, spontaneous saliva swallows were elicited after a short quiet period without explicit countdown. Each participant completed three spontaneous swallows separated by brief rest. During acquisition, the room was quiet, visual distractions were minimized, and participants were instructed to refrain from speaking, head movements, or extraneous orofacial activity. The operator monitored signal quality on screen and repeated any trial affected by artefacts. Raw traces were exported immediately after the session for standardized processing. No dedicated acclimatization swallow was performed. Participants had no history or self-reported symptoms of dysphagia, and spontaneous saliva swallowing in a resting clinical setting was considered unlikely to induce relevant state-dependent performance effects.

Time-resolved swallowing indices were derived from the vertical displacement–time trace using a prespecified algorithm. The opening/closing time (OCT) was defined from the first upward deviation exceeding the resting noise band to the subsequent return to occlusal closure identified by zero-crossing of vertical velocity within a tolerance around baseline. The stabilization time (STT) was defined as the interval from the moment of closure to attainment of a stable intercuspal plateau, operationalized as the first epoch of consecutive samples in which vertical displacement and velocity remained within narrow thresholds for a minimum duration. Total swallowing time (SWT) was measured from the onset of opening to the end of stabilization. For each participant, three swallows were processed; the median value across valid trials was retained a priori to reduce the influence of outliers. All parameterization rules and thresholds were fixed before inspecting group labels and were applied automatically with visual verification by a trained analyst.

Two clinically meaningful signs were assessed alongside the kinematic metrics. Tongue thrust (TT) was recorded as present when visible tongue tissue was interposed between the dental arches at the moment of occlusal contact during spontaneous swallow. TT classification was supported by computerized kinesiographic recording, which permits identification of lack of immediate occlusal contact during the initial phase of swallowing compared with voluntary dental intercuspation, rather than relying exclusively on visual inspection. TT was scored at the participant level based on repeated spontaneous swallows. When uncertainty occurred, trials were repeated; persistent uncertainty was coded as missing rather than forced classification. TT scoring was performed by a single trained clinician following predefined operational criteria and a standardized protocol.

Tubal insufficiency (TI) was recorded as present when predefined clinical criteria consistent with tubal insufficiency were met by immittance testing (Clarinet; Inventis SRL, Padua, Italy) conducted the same day as kinesiography. TI was recorded when a type C tympanogram (negative middle-ear pressure) was observed in at least one ear, in the presence of a normal Valsalva maneuver. All participants also underwent pure-tone audiometry prior to the kinesiographic session, confirming hearing thresholds within normal limits from 250 to 8000 Hz (≤25 dB HL). Immittance testing and audiometry were conducted once before swallowing acquisition, and no continuous middle-ear measurements were performed during swallowing. TI classification was based on unilateral or bilateral presence of the above criteria.

For both variables, the examiner used a structured case report form with explicit yes/no options and immediate on-site data entry. Whenever the observation was uncertain, the trial was repeated; persistent uncertainty was coded as missing rather than forced classification.

Quality control procedures were instituted at different levels. At acquisition, the operator verified calibration stability and repeated any swallow that failed real-time quality checks. At processing, automated routines flagged implausible for blinded re-review. The analyst extracting kinematic metrics was blinded to tinnitus status; the clinician recording TT and TI was not blinded to tinnitus status because evaluations were performed within the same session but followed a standardized protocol and did not have access to interim statistical summaries.

Finally, the primary outcomes were OCT and STT, selected a priori as phase-specific indices of neuromuscular coordination and occlusal settling. Total SWT was treated as a secondary continuous outcome. TT and TI were prespecified as secondary categorical outcomes to provide convergent, clinically interpretable signs pertinent to oropharyngeal function and middle-ear aeration.

### Statistical Analysis

The cases suffering tinnitus and the control group were compared on three continuous outcomes (OCT, STT, and SWT) and on two dichotomous variables (TT and TI), while sex was treated as a covariate. For continuous data, central tendency and dispersion within each group were summarized using mean, standard deviation (SD), median, interquartile range (IQR), and range (Min-Max). Categorical variables were summarized as counts and percentages. Distributional assumptions were examined in cases and controls separately with the Shapiro–Wilk test. Whenever at least one group departed from normality, between-group comparisons were performed with the two-sided Mann–Whitney U test. For nonparametric comparisons two effect sizes were reported: the rank-biserial correlation (*r*) and the Hodges–Lehmann estimator (Δ). Dichotomous variables were analyzed using 2 × 2 contingency tables and two-sided Fisher’s exact tests. The association was tested as odds ratios (OR) with 95% confidence intervals (95% CIs). Additionally, risk ratios (RR) and risk differences (RD) with 95% CIs were also reported.

Further, co-occurrence of TT and TI and their relationships with OCT, STT, and SWT were assessed separately within tinnitus cases and controls. Association TT/TI was evaluated in 2 × 2 tables using two-sided Fisher’s exact test; effect size was expressed as OR with 95% CI and complemented by the phi coefficient (*φ*). For TT/TI versus continuous outcomes, two-sided Mann–Whitney tests were used, with effects summarized as the Hodges–Lehmann median difference (Positive–Negative; TT+ or TI+ minus TT−/TI−) and *r* derived from the Mann–Whitney AUC (area under curve); Spearman’s *ρ* provided a monotonic association check.

To address potential confounding arising from the imbalance in age and sex between cases and controls, additional multivariable models were fitted with group (tinnitus vs. control) as the exposure and age and sex as covariates. For continuous outcomes (OCT, STT, SWT), linear regression models with heteroskedasticity-robust standard errors were used to estimate adjusted mean differences (β coefficients) with 95% confidence intervals (CIs). For TT, multivariable logistic regression adjusted for age and sex was applied to estimate adjusted ORs with 95% CIs. For TI, complete separation (0 events in controls) was present; therefore, penalized logistic regression using Firth’s correction was employed to obtain bias-reduced adjusted ORs and corresponding 95% CIs. As a sensitivity analysis, the association between tinnitus status and continuous outcomes was re-estimated within sex strata (female and male samples) with adjustment for age.

Power and sample-size considerations were conducted with methods appropriate to the scale of measurement and were reported for descriptive context only, whereas interpretation of the findings primarily relies on effect sizes and confidence intervals. For the Mann–Whitney comparisons, observed post hoc power at α = 0.05 was estimated. For dichotomous outcomes, power and required sample sizes were estimated from the observed difference in proportions using Cohen’s h formulation. All tests were two-tailed, statistical significance was set at *p* < 0.05 and analyses were carried out by Stata software (v19; StataCorp, College Station, TX, USA).

Finally, to assess whether cases and controls differed collectively across the outcomes, a non-parametric omnibus test that combines the evidence from their individual Mann–Whitney comparisons was executed. Specifically, Fisher’s method to aggregate the two-sided *p*-values into a single statistic, with statistical significance evaluated by a permutation reference distribution obtained through synchronized random relabeling of the case–control indicator across all subjects and variables.

## 3. Results

The sample included 77 tinnitus cases (47 M/30 F; mean age 51.1 ± 14.8 yo) and 78 controls (14 M/63 F; mean age 41.1 ± 18.4 yo).

Given the imbalance in age and sex between tinnitus cases and controls, the unadjusted non-parametric comparisons were complemented with multivariable models adjusting for age and sex to assess the robustness of the observed between-group differences.

Continuous outcomes displayed non-normality in at least one group, supporting the use of nonparametric tests. For OCT, cases showed a mean of 1.751 s (SD 0.922 s) and a median of 1.60 s (IQR 1.10 s), whereas controls had a mean of 1.116 s (SD 0.623 s) and a median of 1.20 s (IQR 0.773 s). The Mann–Whitney comparison indicated a clear difference between groups (*p* < 0.001). The effect size was moderate, with a *r* of approximately 0.402 favoring higher values in cases and a Δ of +0.60 s (tinnitus−controls). STT exhibited a similar pattern. Tinnitus cases had a mean of 1.438 s (SD 0.882 s) and a median of 1.40 s (IQR 1.20 s), whereas controls had a mean of 0.842 s (SD 0.617 s) and a median of 0.72 s (IQR 0.80 s). The Mann–Whitney test again supported a statistically significant difference (*p* < 0.001), with a *r* of 0.419 and a Δ of +0.60 s, indicating longer stabilization among affected patients. SWT showed a smaller, non-significant separation. Tinnitus group had a mean of 2.750 s (SD 0.693 s) and a median of 2.70 s (IQR 0.60 s). The Mann–Whitney result did not reach significance (*p* = 0.115), with a small *r* (0.146) and a Δ of +0.18 s. Results are summarized in Table 1. These findings were confirmed after adjustment for age and sex, with OCT and STT remaining significantly longer in tinnitus cases, whereas SWT remained non-significant.

After adjustment for age and sex, the between-group differences in phase-specific metrics remained robust. The adjusted mean difference for OCT was +0.72 s (95% CI, +0.43 to +1.00; *p* < 0.001) and for STT was +0.61 s (95% CI, +0.32 to +0.89; *p* < 0.001). In contrast, SWT remained non-significant after adjustment (adjusted mean difference +0.13 s; 95% CI, −0.10 to +0.36; *p* = 0.278). In sex-stratified sensitivity analyses adjusting for age within each stratum, the direction of effects for OCT and STT was consistent in females and males, although precision was reduced in the male stratum due to the smaller number of male controls.

For dichotomous outcomes, TT was more frequent in cases than controls, occurring in 14 of 77 cases (18.2%) and 5 of 78 controls (6.4%). Fisher’s exact test was significant (*p* = 0.029). The OR was 3.05 with a 95% CI from 1.08 to 8.61; the corresponding RR was 2.67 (95% CI: 1.05 to 6.77) and the RD was +11.7% (95% CI: 1.3% to 22.4%). After adjustment for age and sex, TT remained associated with tinnitus status (adjusted OR 4.07; 95% CI, 1.14 to 14.55; *p* = 0.031). TI was observed in 13 of 77 cases (16.9%) and in none of the 78 controls, yielding a significant Fisher’s exact test (*p* < 0.001). The corrected OR was 32.85 (95% CI: 1.92 to 563.49), with a RD of +16.9% (95% CI: 8.6% to 26.8%) and a correspondingly large RR (27.35), reflecting the zero prevalence in controls. Using Firth-penalized logistic regression adjusted for age and sex, TI remained markedly more frequent in cases (adjusted OR 35.49; 95% CI, 2.15 to 587.21; *p* = 0.013), with wide CIs reflecting sparse-data uncertainty (Table 2). These findings show that TT forward against or between the teeth during swallowing and TI are more frequent in tinnitus patients, the latter not being observed at all among controls.

Within tinnitus cases (n = 77), TT and TI co-occurred in 2 participants (2.6%), with no evidence of dependence (OR = 0.79, 95% CI 0.15–4.03; Fisher’s *p* = 1.00; *φ* = −0.03). In controls (n = 78), TI was not observed, precluding a meaningful estimate of TT–TI association. In tinnitus cases, TT+ subjects (n = 14) showed clearly prolonged OCT compared with TT− (2.22 vs. 1.45 s; Δ +0.88 s; *r* = 0.60; Mann–Whitney *p* ≤ 0.001), whereas STT and SWT were only minimally and non-significantly affected (Δ ≤ +0.20 s; *r* ≤ 0.18; *p* ≥ 0.60). TI status was not materially related to OCT, STT, or SWT in tinnitus cases (Δ ≤ +0.20 s; *r* ≤ 0.13; *p* ≥ 0.46), and in controls TT was infrequent (5/78) and showed no systematic association with any swallowing-time parameter (Mann–Whitney *p* ≥ 0.59; *r* ≤ 0.15), while TI remained absent. Spearman analyses confirmed a moderate positive monotonic association between TT and prolonged OCT in tinnitus patients (*ρ* = 0.40, *p* < 0.001), while all other correlations between TT/TI and swallowing-time parameters were very small (*ρ* ≤ 0.12, *p* ≥ 0.30) and clinically negligible. In synthesis, these analyses indicate that TT in tinnitus patients is specifically associated with a marked prolongation of OCT, while STT and total SWT remain essentially unchanged. TI and TT do not meaningfully co-occur, and TI alone shows no relevant impact on swallowing timing parameters.

Power estimates are reported for descriptive context only, whereas interpretation of the findings primarily relies on effect sizes and confidence intervals. The observed power for OCT and for STT were consistent with the magnitude of the detected effects for OCT and STT, while SWT showed limited sensitivity at the current sample size. The sample size per group needed to attain 80% power would exceed the current allocation. For context on the dichotomous variables, the observed effect sizes corresponded to high power for TT (0.867) and moderate power for TI (0.745), consistent with the significant Fisher tests despite the sparse counts in the latter.

The omnibus combination test indicated a clear global separation between groups when the three continuous outcomes were considered jointly. The Fisher combined statistic was 50.40, and the permutation-based *p*-value was significant (<0.001), confirming that cases and controls differ overall across OCT, STT, and SWT, despite the latter showing only a modest univariate effect. Overall, age/sex-adjusted analyses yielded results that were concordant with unadjusted comparisons, supporting the robustness of the association between tinnitus status and prolonged phase-specific swallowing timings (OCT and STT).

## 4. Discussion

The present study delineates a coherent swallowing-related profile in adults with tinnitus, characterized by relative prolongation of the opening/closing phase and the post-closure stabilization phase, along with a higher prevalence of tongue thrust and clinical signs consistent with TI. Collectively, these findings suggest altered coordination at the interface where mandibular re-closure, tongue withdrawal, palatal activity, and middle-ear aeration must be tightly synchronized.

Swallowing is generated by a brainstem central pattern generator and refined by cerebellar and corticobulbar inputs [18]. During a saliva swallow, jaw depressors initiate opening, the tongue gathers and shapes the saliva bolus against the palate, jaw elevators return the mandible to intercuspation, and a short stabilization interval follows as the system settles into a low-velocity, steady state [19]. This sequence depends on dense somatosensory feedback from periodontal mechanoreceptors, muscle spindles of the jaw closers and openers, mucosal receptors, and tongue-palate contact [20]. A prolonged OCT suggests hesitant or energetically inefficient re-closure, while prolonged STT indicates a slower attainment of a quiet intercuspal plateau after initial contact [21]. Both phenomena are compatible with altered sensorimotor gain or compensatory tongue behaviors that delay the final handoffs of the oral phase.

The trigeminal system provides a unifying anatomical substrate for these effects [22,23]. The mandibular division supplies the principal jaw elevators and depressors and innervates the tensor veli palatini, which dilates the Eustachian tube during swallowing. Consequently, the same neural drive that coordinates mandibular re-closure and stabilization also supports palatal activity and middle-ear pressure regulation. If mandibular re-closure is imprecise, the palatal–Eustachian maneuver is more likely to be mistimed or underpowered. Inefficient tube opening would then manifest clinically as tubal insufficiency, while the mandibular kinematic record would show delayed stabilization [9]. The concurrence of these signs in tinnitus aligns with long-standing models of somatosensory influence on auditory function, in which craniofacial inputs shape the excitability of auditory brainstem circuits [2].

The tongue occupies an important position in this coordination. In a typical saliva swallow, the dorsum and tip contact the palate in a stereotyped sequence, then withdraw as the mandible returns to a reproducible intercuspal position [24]. Mechanically, the TT can obligate micro-corrections in mandibular position, thereby prolonging the OCT; as the tongue retreats, the mandible may oscillate briefly before reaching a stable plateau, prolonging the stabilization interval. Neurophysiologically, persistent interposition implies that the brainstem pattern is compensating for altered afferent feedback or for a shifted motor set, resulting in a swallow that is prolonged in time [25].

Eustachian tube mechanics further reinforce the previous interpretations. During a normal swallow, tensor and levator veli palatini coordinate to open the cartilaginous tube and equalize middle-ear pressure [26]. Suboptimal activation, whether due to insufficient drive, delayed timing, or interference from concurrent mandibular adjustments, can result in incomplete or inconsistent tube opening [9]. Clinically, TI may represent a relevant correlate and a potential contributor to the variability in tinnitus loudness or quality. The combination of delayed mandibular stabilization and signs of suboptimal tube opening is consistent with a broader alteration of the integrated orofacial–palatal program, rather than an isolated jaw phenomenon even though TI was not independently associated with kinematic parameters in this sample.

In synthesis, phase-resolved metrics are physiologically informative. Total swallowing time aggregates multiple sub-events, which can dilute between-group contrasts. Opening/closing and stabilization capture the key transitions into occlusal contact and stable rest. These intervals rely on proprioception and damping of residual motion and are sensitive to small changes in occlusal feedback, tongue posture, and muscle tone. Hence, phase-specific measures may separate groups better than the global duration.

Consistent with these patterns, the correlation analyses showed that only the OCT was meaningfully linked to TT in tinnitus cases. The moderate positive correlation between TT and OCT, together with negligible correlations for STT, SWT and for TI, indicates that alterations in tongue posture primarily destabilize the onset of the swallow rather than its overall duration or post-closure stabilization. This pattern supports the idea that a disrupted tongue–mandible–palate interface at swallow onset is a key somatosensory contributor in this subgroup, whereas TI per se is not mirrored by measurable changes in mandibular kinematics.

The identification of altered mandibular swallowing kinematics may contribute to recognizing patients with a somatosensory component of tinnitus, potentially improving phenotypic stratification. As reported by Ralli et al. temporomandibular or cervical dysfunctions are significantly associated with tinnitus and may represent modifiable contributors in selected subgroups [27].

Myofunctional approaches that normalize tongue posture and sequencing may reduce interposition and facilitate a brisk, unobstructed return to intercuspation [28]. Occlusal strategies that restore predictable contacts and periodontal feedback may help the system terminate closure with fewer micro-corrections and dampen residual oscillations more rapidly [18]. Palatal and Eustachian-tube–oriented rehabilitation, embedded within swallowing tasks, may strengthen and retime the dilation maneuver that equalizes middle-ear pressure. The common objective across these modalities is not merely to shorten the swallow but to restore crisp, well-timed transitions that minimize aberrant somatosensory inflow to auditory pathways [2,8,22]. These interventional findings remain heterogeneous and context-specific and cannot be extrapolated to the present observational results.

Finally, the frequency of swallowing in daily life provides a compelling rationale for why relatively small inefficiencies might matter. Even minor delays, repeated hundreds of times per day, can consolidate as habitual motor strategies and sustain a steady somatosensory drive to auditory-connected nuclei. In this view, the swallowing signature observed in tinnitus may represent a concomitant physiological finding in tinnitus. Recognizing and addressing the shared orofacial–palatal substrate may therefore be a useful step toward mechanism-informed management of tinnitus in selected patients.

### Limitations

TT and TI assessments were not fully blinded, and no formal inter-rater reliability evaluation was performed, which may introduce potential observer bias and should be considered when interpreting these findings. Moreover, given the demographic imbalance between tinnitus cases and controls, even though the multivariable analyses adjusted for age and sex showed results consistent with the primary findings, residual confounding inherent to the observational design cannot be fully excluded.

## 5. Conclusions

Adults with tinnitus exhibit a distinct kinematic profile of saliva swallowing, characterized by moderately prolonged OCT and post-closure STT, whereas SWT differs only slightly from tinnitus-free controls and does not reach statistical significance. In parallel, TT and TI are both more prevalent in tinnitus patients than in controls, although TI is not accompanied by measurable changes in OCT, STT, or SWT and TT and TI do not meaningfully co-occur.

Taken together, these findings indicate that tinnitus is associated with phase-specific inefficiencies of mandibular re-closure and stabilization and with an increased burden of functional signs linked to tongue posture and Eustachian-tube function. Among these, TT in tinnitus patients shows a specific and clinically relevant association with a marked prolongation of OCT, while STT and SWT remain largely unchanged, and TI does not exert a detectable impact on mandibular kinematics.

The present findings should be interpreted strictly as exploratory physiological associations within a complex and multifactorial condition. Future research should confirm these associations in larger samples with adjusted analyses, incorporate additional physiological correlates.

## Figures and Tables

**Table 1:** Swallowing timing metrics in participants with tinnitus versus controls. Values are mean ± SDs. Two-sided Mann–Whitney tests are reported with the rank-biserial correlation (*r*) and the median difference (Δ tinnitus-controls); positive Δ indicates longer times in the tinnitus group.

Outcome	Mean ± SD Tinnitus	Mean ± SD Controls	*p*-Value	*r*	Δ Tinnitus-Controls	Δ Tinnitus-Controls Adjusted	Adjusted *p*-Value
OCT	1.751 ± 0.922	1.116 ± 0.623	<0.001	0.402	+0.60	+0.72 s	<0.001
STT	1.438 ± 0.882	0.842 ± 0.617	<0.001	0.419	+0.60	+0.61 s	<0.001
SWT	2.750 ± 0.693	2.570 ± 0.650	0.115	0.146	+0.18	+0.13 s	0.278

**Table 2:** Dichotomous outcomes in tinnitus cases and controls, unadjusted and adjusted for age and sex.

Outcome	Tinnitus (n = 77)	Controls (n = 78)	Fisher *p*	OR Unadjusted	95% CI	OR Adjusted	95% CI	Adjusted *p*-Value
TT	14 (18.2%)	5/78 (6.4%)	0.029	3.05	1.08–8.61	4.07	1.14–14.55	0.031
TI	13 (16.9%)	0/78 (0.0%)	<0.001	32.86	1.92–563.46	35.49	2.15–587.21	0.013

## Data Availability

The data presented in this study are available on request from the corresponding author due to ethical restrictions.

## Abbreviations

The following abbreviations are used in this manuscript:

OCT	Opening/close time
STT	Stabilization time
SWT	Swallowing time
TI	Tubal insufficiency
TT	Tongue thrust

## References

[1] Jarach C.M., Lugo A., Scala M., Van Den Brandt P.A., Cederroth C.R., Odone A., Garavello W., Schlee W., Langguth B., Gallus S. (2022). Global Prevalence and Incidence of Tinnitus: A Systematic Review and Meta-analysis. JAMA Neurol..

[2] Shore S.E., Wu C. (2019). Review Mechanisms of Noise-Induced Tinnitus: Insights from Cellular Studies. Neuron.

[3] Li Y., Yang H., Niu X., Sun Y. (2024). The Long-Term Effect of Cochlear Implantation on Tinnitus: A Systematic Review and Meta-Analysis. Diagnostics.

[4] Dehmel S., Cui Y.L., Shore S.E. (2008). Cross-modal interactions of auditory and somatic inputs in the brainstem and midbrain and their imbalance in tinnitus and deafness. Am. J. Audiol..

[5] Sanchez T.G., Rocha C.B. (2011). Diagnosis and management of somatosensory tinnitus: Review article. Clinics.

[6] Teixeira M.S., Banks J., Swarts J.D., Alper C.M., Doyle W.J. (2014). Eustachian Tube Opening Measured by Sonotubometry is Poorer in Adults with a History of Past Middle Ear Disease. Int. J. Pediatr. Otorhinolaryngol..

[7] Steele C.M., Miller A.J. (2010). Sensory input pathways and mechanisms in swallowing: A review. Dysphagia.

[8] Conlon B., Langguth B., Hamilton C., Hughes S., Meade E., Connor C.O., Schecklmann M., Hall D.A., Vanneste S., Leong S.L. (2020). Bimodal neuromodulation combining sound and tongue stimulation reduces tinnitus symptoms in a large randomized clinical study. Sci. Transl. Med..

[9] Schilder A.G.M., Bhutta M.F., Butler C.C., Holy C., Levine L.H., Kvaerner K.J., Norman G., Pennings R., Poe D., Silvola J. (2015). Eustachian tube dysfunction: Consensus statement on definition, types, clinical presentation and diagnosis. Clin. Otolaryngol..

[10] Jackson R., Vijendren A., Phillips J. (2019). Objective Measures of Tinnitus: A Systematic Review. Otol. Neurotol..

[11] Michiels S. (2023). Somatosensory Tinnitus: Recent Developments in Diagnosis and Treatment. J. Assoc. Res. Otolaryngol..

[12] Telischi J., Rossborough J., Kuzbyt B., Rajguru S.M., Snapp H.A., Scaglione T. (2025). A Systematic Review of Psychometric Validation for Subjective Tinnitus Outcome Measures Assessing Acute Treatment Effects. Otol. Neurotol. Open.

[13] De La Torre Canales G., Christidis N., Grigoriadis A., Strandberg T., Montan V., Medina Flores D., Al-Moraissi E.A., Christidis M. (2025). Associations between temporomandibular disorders and tinnitus—A systematic review. Cranio.

[14] McFerran D.J., Stockdale D., Holme R., Large C.H., Baguley D.M. (2019). Why is there no cure for tinnitus?. Front. Neurosci..

[15] Von Elm E., Altman D.G., Egger M., Pocock S.J., Gøtzsche P.C., Vandenbroucke J.P. (2007). Strengthening the reporting of observational studies in epidemiology (STROBE) statement: Guidelines for reporting observational studies. BMJ.

[16] World Medical Association (2013). World Medical Association Declaration of Helsinki: Ethical principles for medical research involving human subjects. JAMA.

[17] Jankelson B., Swain C.W., Crane P.F., Radke J.C. (1975). Kinesiometric instrumentation: A new technology. J. Am. Dent. Assoc..

[18] Jean A. (2001). Brain stem control of swallowing: Neuronal network and cellular mechanisms. Physiol. Rev..

[19] Bourdiol P., Mishellany-Dutour A., Peyron M.A., Woda A. (2014). Tongue-mandible coupling movements during saliva swallowing. J. Oral Rehabil..

[20] Trulsson M. (2006). Sensory-motor function of human periodontal mechanoreceptors. J. Oral Rehabil..

[21] Yaşaroǧlu Ö.F., Arslan S.S., Cengiz E., Alici R., Demir N., Oǧuz B., Düger T. (2024). Swallowing kinematics and submental muscles activation during a newly designed maneuver called Mouth Open Swallowing Maneuver: A comparative study. PLoS ONE.

[22] Shore S., Zhou J., Koehler S. (2007). Neural mechanisms underlying somatic tinnitus. Prog. Brain Res..

[23] Bičanić I., Hladnik A., Džaja D., Petanjek Z. (2019). The anatomy of orofacial innervation. Acta Clin. Croat..

[24] Álvarez G., Dias F.J., Lezcano F., Arias A., Navarro P., Fuentes R. (2020). Description of tongue movements on swallowing patterns. Arch. Oral Biol..

[25] Matsuo K., Palmer J.B. (2008). Anatomy and Physiology of Feeding and Swallowing—Normal and Abnormal. Phys. Med. Rehabil. Clin. N. Am..

[26] Szymanski A., Agarwal A. (2023). Anatomy, Head and Neck, Ear Eustachian Tube.

[27] Ralli M., Altissimi G., Turchetta R., Mazzei F., Salviati M., Cianfrone F., Orlando M.P., Testugini V., Cianfrone G. (2016). Somatosensory Tinnitus: Correlation between Cranio-Cervico-Mandibular Disorder History and Somatic Modulation. Audiol. Neurootol.

[28] Shah S.S., Nankar M.Y., Bendgude V.D., Shetty B.R. (2021). Orofacial Myofunctional Therapy in Tongue Thrust Habit: A Narrative Review. Int. J. Clin. Pediatr. Dent..

